# The Gut Microbiome of Children during the COVID-19 Pandemic

**DOI:** 10.3390/microorganisms10122460

**Published:** 2022-12-13

**Authors:** Mickayla Bacorn, Hector N. Romero-Soto, Shira Levy, Qing Chen, Suchitra K. Hourigan

**Affiliations:** Clinical Microbiome Unit (CMU), Laboratory of Host Immunity and Microbiome (LHIM), National Institute of Allergy and Infectious Diseases (NIAID), National Institutes of Health (NIH), Bethesda, MD 20892, USA

**Keywords:** SARS-CoV-2, microbiota, children, immune education, gut barrier integrity, hygiene hypothesis

## Abstract

The gut microbiome has been shown to play a critical role in maintaining a healthy state. Dysbiosis of the gut microbiome is involved in modulating disease severity and potentially contributes to long-term outcomes in adults with COVID-19. Due to children having a significantly lower risk of severe illness and limited sample availability, much less is known about the role of the gut microbiome in children with COVID-19. It is well recognized that the developing gut microbiome of children differs from that of adults, but it is unclear if this difference contributes to the different clinical presentations and complications. In this review, we discuss the current knowledge of the gut microbiome in children with COVID-19, with gut microbiome dysbiosis being found in pediatric COVID-19 but specific taxa change often differing from those described in adults. Additionally, we discuss possible mechanisms of how the gut microbiome may mediate the presentation and complications of COVID-19 in children and the potential role for microbial therapeutics.

## 1. Introduction

In the COVID-19 pandemic, caused by the SARS-CoV-2 virus, over 600 million people have been infected, and over 6 million deaths have occurred globally [1]. Although clinical studies have revealed that adults and immunocompromised patients present with more severe disease, COVID-19 has also significantly impacted the pediatric population [2,3]. While the literature shows that the prevalence of pediatric COVID-19 ranges from 1 to 13.3% of the total cases [4], it is suggested that this is underestimated. As many pediatric cases are asymptomatic or mild, testing is not performed, and therefore these cases are not documented. Due to the differences in overall severity and underreporting of cases, there are fewer pediatric studies related to COVID-19, but these studies are important as children can be severely affected [5,6] and can suffer from long-term health outcomes [7].

Moreover, there is growing data supporting the role of the gut microbiome in disease severity, immunological dysfunction, and long-term outcomes in adults with COVID-19 [2,8,9]. There is a paucity of such data in children, which is important to study for several reasons. Firstly, in addition to having a different clinical presentation of COVID-19 than adults [10], children also have different immune responses and a developing gut microbiome, which differs from the adult gut microbiome [11,12,13]. Characterizing the role of the underlying gut microbiome in pediatric COVID-19 may explain the differences in pathogenesis and clinical presentation between children and adults. In addition, the rapid dynamic development of the gut microbiome in young children is associated with immune education, with disruptions during this critical window having long-term immune and inflammatory health consequences [14,15]. Understanding how host-microbiome responses may be disrupted during COVID-19 infection in early childhood may give insight into some of the complications and long-term health outcomes of COVID-19 that are more unique to children, such as multisystem inflammatory syndrome in children (MIS-C), and also into a potential role for microbial therapeutics [16]. Furthermore, the change in environmental exposures during the pandemic may potentially impact the early life developing microbiome with subsequent health effects [17]. 

Therefore, in this review, the following is discussed: (1) the role of the pediatric gut microbiome in COVID-19 from prenatal exposure through childhood and in long-term outcomes; (2) the underlying mechanisms by which the pediatric microbiome may modulate the presentation of COVID-19 in children; and (3) the potential role for microbiome therapeutics for COVID-19 in children. 

## 2. The Gut Microbiome in Different Presentations of COVID-19 in Children

### 2.1. Asymptomatic and Symptomatic Children with COVID-19

COVID-19 infections have a wide range of presentations and are classified clinically as asymptomatic, mild, moderate, severe, or critical [18]. Overall, children have a less severe presentation of COVID-19 than adults [2]. Interestingly, while children with COVID-19 present with typical respiratory symptoms, 15–84% of children with COVID-19 have at least one GI symptom [19], such as vomiting, diarrhea, and abdominal pain, all of which are symptoms associated with alterations in the microbiome [20]. The variation in severity and symptomatic presentation complicates identification, containment, treatment, and research as asymptomatic patients have no symptoms of disease, while others experience severe symptoms progressing up to hospitalization, mechanical ventilation in an intensive care unit (ICU), or even death [21]. Limiting the spread of COVID-19 is challenging because, although it is hard to accurately measure, it is predicted that 5–24% of cases are asymptomatic but still infectious [22]. The literature is unclear regarding pediatric asymptomatic prevalence, and the data ranges from 1 to 35% [4,23,24,25]. One study looking at SARS-CoV-2 seroprevalence in children found that 66% of positive children had never had symptoms of COVID-19 [26]. Furthermore, a multi-hospital analysis (*n* = 438) found that even extensive symptom-based testing protocols failed to identify 45% of pediatric COVID-19 amongst hospitalized children [27]. Thus, the prevalence of asymptomatic children with COVID-19 is unclear, and the full impact in children remains unknown. 

Minimal data exist regarding the gut microbiome in children with asymptomatic COVID-19. Utilizing the fact that SARS-CoV-2 has been detected in children’s stool for a prolonged period of up to 14 days beyond a negative nasal swab [28,29], one study early in the pandemic examined SARS-CoV-2 in longitudinal stool samples of infants [30]. They detected SARS-CoV-2 in the stool of one infant before the first confirmed case in the region, indicating the undetected presence of COVID-19. The asymptomatic infants with SARS-CoV-2 in their stool (*n* = 13 COVID-19, *n* = 26 uninfected matched controls) had decreases in the following bacteria: *Bifidobacterium bifidum*, *Akkermansia muciniphila*, *Eubacterium limosum*, *Enterocloster clostridioformis*, *Blautia hominis*, *Veillonella dispar*, and *Enterobacter cloacae* (Table 1). Of particular importance, *Bifidobacterium bifidum* and *Akkermansia muciniphila* are known anti-inflammatory bacterial taxa [31,32]. The future impact of these depletions on the infant microbiome during this critical window of development and subsequent immune and inflammatory responses is unclear. However, this effect deserves further exploration given the risk of developing autoimmune and autoinflammatory conditions in children with COVID-19 [33].

In adults, microbiome dysbiosis in relation to disease severity of COVID-19 has been relatively well studied. Although gut microbiome dysbiosis can be seen even in those with asymptomatic or mild disease [34], more severe gut microbiome dysbiosis and depletion of beneficial taxa were observed in those with more severe disease [8]. Moreover, this dysbiosis correlated with higher levels of inflammatory cytokines in patients with COVID-19, suggesting that the gut microbiome is involved in the magnitude of COVID-19 severity, possibly via modulation of the host immune response. Furthermore, microbiome dysbiosis persisted even after disease recovery [8].

Children generally have less severe symptoms of COVID-19 than adults, but severe disease can occur [5,6]. There is less known regarding the degree of gut microbiome dysbiosis in children relating to disease severity during COVID-19 infection. This is important to study as gastrointestinal (GI) symptoms are commonly found in children, correlate with disease severity, and are often associated with gut microbiome changes [19,20,35,36,37]. Romani et al. studied the gut microbiome in children presenting with different severities of COVID-19 (*n* = 7 moderate, 49 mild, 12 asymptomatic) [38]. They found that moderate cases had lower α-diversity than mild cases, and that mild cases had lower α-diversity than asymptomatic cases (*p* < 0.05) (Table 1). They also saw a trend towards individual clusters for β-diversity between groups.

Given the frequency of asymptomatic cases, the pediatric population presents an interesting opportunity to analyze the microbiome differences between asymptomatic and symptomatic disease progression. Symptomatic presentation may be correlated with decreases in microbiome diversity, and even asymptomatic presentation may be associated with decreased abundance of anti-inflammatory bacteria [30,38]. In the future, research expanding these findings may provide insight into why the pediatric population in general has less severe disease compared to adults.

**Table 1:** Current research on pediatric gut microbiota changes in relation to COVID-19 infection analyzed via 16S ribosomal RNA gene sequencing of stool samples.

Title	Author, Location, Date Published	Patients	Ages	α-Diversity	β-Diversity	Enriched Bacteria	Reduced Bacteria	Key Findings
Progressive deterioration of the upper respiratory tract and the gut microbiomes in children during the early infection stages of COVID-19 [39]	Xu R et al. Shanghai, China September 2021	9 COVID-19, 9 healthy controls	7 months–12 years	Significant between some gut clusters and healthy mix of upper respiratory and gut community	N/A	COVID-19 *: Bacteroidetes, Firmicutes, *Pseudomonas*, *Herbaspirillum*, *Burkholderia*, *Pseudomonas veronii*, *Streptococcus*	N/A	Dysbiosis was sustained for 25–58 days Clustering gut composition revealed stepwise dysbiosis
Gut microbiota changes are detected in asymptomatic very young children with SARS-CoV-2 infection [30]	Nashed L et al. Falls Church, VA, USA February 2022	595 participants (13 participants had COVID-19, 26 matched controls)	Newborn–2 years	Not significant	Not significant	N/A	COVID-19 *: *Bifidobacterium bifidum*, *Veillonella dispar*, *Enterobacter cloacae*, *Akkermansia muciniphila*, *Eubacterium limosum*, *Enterocloster clostridioformis*, *Blautia hominis*	SARS-CoV-2 was present in infant stool before first reported case
Intestinal microbiota composition of children with infection with severe acute respiratory syndrome coronavirus 2 (SARS-CoV-2) and multisystem inflammatory syndrome (MIS-C) [16]	Suskun C et al. Eskisehir, Turkey May 2022	20 COVID-19, 25 MIS-C, 19 healthy controls	5–11 years	Significant between MIS-C and controls	Significant between MIS-C and controls	COVID-19 *: *Clostridium*, *Bacteroides coprophilus*, *Eubacterium dolichum*, *Bacteroides uniformis*, *Clostridium piliforme* COVID-19 ***: *Bacteroides coprophilus*, *Bifidobacterium adolescentis*, *Dorea formicigenerans*, *Ruminococcus albus*, *Clostridium piliforme* MIS-C *: *Bacteroides*, *Eggerthella*, *Prevotella* *Bacteroides uniformis*, *Bacteroides plebeius*, *Clostridium ramosum*, *Eubacterium dolichum*, *Eggerthella lenta*, *Bacillus thermoaamylovorans*, *Prevotella tannerae*, and *Bacteroides coprophilus* MIS-C **: *Bacteroides*, *Eggerthella*, *Clostridium*, *Bacteroides uniformis*, *Bacillus thermoaamylovorans*, *Eubacterium dolichum*	COVID-19 and MIS-C *: *Faecalibacterium prausnitzii* COVID-19 *: *Eubacterium*, *Roseburia*, *Lachnospiraceae* MIS-C **: Firmicutes	*Faecalibacterium prausnitzii* reduced in COVID-19 and MIS-C cases
The Relationship between pediatric gut microbiota and SARS-CoV-2 infection [38]	Romani L et al. Rome, Italy July 2022	88 suspected COVID-19, 95 healthy controls	8 days–17 years	Significant between COVID-19 and controls	Significant between COVID-19 and controls	COVID-19 *: *Faecalibacterium*, *Fusobacterium*, *Neisseria* MIS-C *: *Veillonella*, *Clostridium*, *Dialister*, *Ruminococcus*, *Streptococcus*	COVID-19 *: *Bifidobacterium*, *Blautia*, *Ruminococcus*, *Collinsella*, *Coprococcus*, *Eggerthella*, *Akkermansia* MIS-C *: *Bifidobacterium*, *Blautia*, *Granulicatella*, *Prevotella*	Moderate COVID-19 had ↓ α-diversity than mild and asymptomatic Enriched *Faecalibacterium* specific to pediatric COVID-19 (not seen in adults)

* Compared to healthy controls. ** Compared to healthy controls and COVID-19 cases. *** Compared to healthy controls and MIS-C cases.

### 2.2. Pregnancy and Neonates

COVID-19 infection during pregnancy may have consequences for the mother and offspring, some of which can be speculated to be partially microbiome-mediated, both directly and indirectly. Most pregnant patients diagnosed with COVID-19 are discharged without any major complications, yet severe symptoms, maternal morbidity, and perinatal deaths have been reported [40,41]. An increased decidual immune response was associated with COVID-19 later during gestation in pregnancy [42]. The gut microbiome is known to drive various inflammatory responses during different gestational stages of pregnancy and could be speculated to play a role in the varying immune responses to COVID-19 observed during the different stages of pregnancy [43]. More longitudinal studies including pregnant women infected with COVID-19 may detect if there are any significant changes in the microbiome composition correlating to alterations in the immune response against the disease. 

There are multiple prenatal and early life factors influencing the development of the infant gut microbiota that may be influenced by COVID-19 during pregnancy. For example, preterm infants are known to have an unstable and undeveloped microbiome, especially when treated with antibiotics [44,45]. Although an overall decrease in the preterm birth rate was seen at the start of the pandemic, pregnant mothers with COVID-19 are more likely to have preterm births, including very early pre-term births, compared to those who are uninfected [46,47,48,49]. Furthermore, COVID-19 infection in pregnancy is associated with an increased risk of delivery by Cesarean section (C-section) [46]. C-section deliveries are known to increase the risk of inflammatory diseases in the offspring, including obesity and atopy, which are hypothesized to develop through a microbiome-mediated mechanism [50,51,52,53,54,55]. Compounding this, antibiotics are frequently prescribed to those with COVID-19, including during pregnancy [56,57]. Prenatal and peripartum antibiotic exposure is also associated with an altered microbiome and increased risk of developing conditions such as obesity and asthma [58,59,60,61], as the majority of microbiome maturation occurs during the first year of life, educating immune and metabolic development [11,13]. 

The effects of breast milk on the infant gut microbiome during the COVID-19 pandemic should also be considered. Breast milk plays a key role in healthy infant gut microbiome development, both through human milk oligosaccharides, which strongly select for a limited repertoire of beneficial organisms in the infant gut, and through its own unique microbiome [62,63]. Breast-milk-induced microbiome changes in the infant gut are believed to impact immune function in early life, with breastfeeding known to reduce the risk of future inflammatory diseases [62,64,65,66]. It is thought that the likelihood of transmitting SARS-CoV-2 from mother to infant through breast milk is low, and current recommendations are that infants should receive breast milk from mothers with COVID-19 following appropriate safety measures for contact [67,68,69]. Moreover, IgA antibodies against SARS-CoV-2 have been detected in breast milk, which may reduce the clinical impact in infants with viral exposure. Despite this, some insecurity may remain regarding this practice in mothers and care providers, potentially reducing the rate of breastfeeding [67,70].

Neonates born to mothers with COVID-19 during pregnancy and in the peripartum period should be followed over time to assess whether any of these factors impact the microbiome development. No current data exists regarding these potential long-term health outcomes yet.

### 2.3. Multisystem Inflammatory Syndrome in Children (MIS-C)

While the majority of children show asymptomatic infection or mild COVID-19, some children develop multisystem inflammatory syndrome in children (MIS-C), a post-infectious syndrome resembling Kawasaki disease [33]. MIS-C is a rare but severe condition involving at least two organ systems with inflammation in previously healthy children under 21 years of age. It occurs 2–4 weeks after infection or exposure to SARS-CoV-2. The symptoms of MIS-C include, but are not limited to, persistent fever, abdominal pain, vomiting, diarrhea, skin rash, mucocutaneous lesions, and, in severe cases, hypotension and shock. Patients with MIS-C have elevated inflammatory markers, such as C-reactive protein (CRP) [71]. GI symptoms are one of the predominant MIS-C symptoms, which suggests that the gut microbial community might serve as local and systematic inflammatory modulators through interactions with SARS-CoV-2 [72]. Moreover, gut barrier dysfunction has been reported in MIS-C [73]. Immunological features in MIS-C have been reported; however, research examining the role of the gut microbiota in the pathogenesis of MIS-C is limited and is important to study given the interplay of the gut microbiome and host in other autoimmune diseases [74,75].

In the Romani et al. study examining the gut microbiome in children with COVID-19, four patients with MIS-C were included [39]. Compared to healthy controls, the gut microbiome in MIS-C showed an increase of *Veillonella*, *Clostridium*, *Dialister*, *Ruminococcus*, and *Streptococcus* and a decrease of *Bifidobacterium*, *Blautia*, *Granulicatella*, and *Prevotella* (Table 1). However, this comparison is limited due to the small sample size. 

In a larger cohort, Suskun et al. analyzed the gut microbiota composition of 64 children (*n* = 25 MIS-C, 20 COVID-19, 19 healthy) [16]. The Shannon index was higher in the MIS-C cohort compared to healthy controls, although there was no difference in other alpha diversity measures (Table 1). A significant difference in beta diversity was also seen between MIS-C and healthy controls. Of note, lower Firmicutes and higher Bacteroides have been reported in Kawasaki disease, which resembles MIS-C symptomatically [76]. Consistently, compared to the healthy control and the COVID-19 groups, an increased abundance of Bacteroidetes and decreased ratio of Firmicutes:Bacteroides was seen in the MIS-C cohort [16]. In children with MIS-C, there was a decrease in the anti-inflammatory taxa and an increase in pro-inflammatory taxa. *Faecalibacterium prausnitzii*, which has been reported to maintain gut physiology and reduce intestinal mucosal inflammation through butyrate [77,78], was underrepresented in children with MIS-C and COVID-19 [16]. Additionally, an increased relative abundance of *Eggerthella lenta*, previously described to be involved in autoimmunity [79,80], and *Eubacterium dolichum*, previously implicated in metabolic dysfunction [81], were observed in children with MIS-C [16]. Proteobacteria have been associated with metabolic disorders and inflammatory bowel disease [82], and an increased abundance was seen in MIS-C (11%) compared to healthy controls (5%) [16]. In summary, the gut microbiota changed dramatically, reflecting an increase in opportunistic pathogens and a depletion of beneficial commensals in MIS-C children. However, a limitation of this study was that the samples were taken at a single time point during the MIS-C illness, and conclusions cannot be drawn as to whether these microbiota changes were a cause or consequence of the inflammation observed in MIS-C. Longitudinal studies are needed to elucidate the cause-effect relationship between microbiota alteration and symptomatology seen in MIS-C, which are difficult to conduct given that MIS-C is a rare consequence of COVID-19 and it is unclear which children will develop it.

### 2.4. Long-Term Health Consequences

Post-acute COVID-19 syndrome or “long COVID” is characterized by persistent symptoms after COVID-19 infection [83,84]. This has been reported in children, with the most common features being fatigue and mood symptoms [85]. In adults, prolonged gut microbiome dysbiosis has been associated with long COVID, with higher levels of *Ruminococcus gnavus* and *Bacteroides vulgatus* and lower levels of *Faecalibacterium prausnitzii* [9]. To our knowledge, the gut microbiome has not yet been studied in children with long COVID but doing so may help to gain insight into why some children have prolonged symptoms. 

From the limited number of studies available, there is evidence of gut microbiome dysbiosis in children with both asymptomatic and symptomatic COVID-19, in addition to MIS-C. However, specific microbiome changes vary between studies, likely from differences in methodologies and cohorts. High-quality, large, controlled longitudinal studies are needed to further elucidate the role of the gut microbiome in children with COVID-19 and how it may influence long-term health outcomes.

### 2.5. The Gut Microbiome and Different Variants of COVID-19

While there are thousands of documented SARS-CoV-2 mutations, only some have made a significant impact throughout the pandemic. The Center for Disease Control and Prevention (CDC) has classified 12 variants of interest or concern that have circulated or are currently circulating in the US population [86]. Although there is high similarity between all variants of SARS-CoV-2, some of the variants have increased infectivity compared to the initial wild-type virus [87]. This complicates the pandemic, as certain treatments and vaccines work for some variants but are ineffective against others [88]. Moreover, different variants seem to have different clinical presentations in children. For example, there was an increase in hospitalization rates for 5- to 11-year-olds during the predominance of the Omicron variant [89], although the disease progression was less severe compared to the hospitalizations observed during the Delta-predominant period.

An in silico study simulated interactions between metabolites and natural products of commensal bacteria and the receptor-binding domain (RBD) of the SARS-CoV-2 spike protein. Interestingly, specific bile acids and non-ribosomal peptides may have sufficient affinity to bind to the RBD of SARS-CoV-2 spike glycoproteins and reduce infectivity [90]. However, newer variants may include an amino acid mutation that reduces this affinity, rendering the metabolites and natural products of commensal bacteria ineffective against the virus. This suggests that the microbial products of gut bacteria may put selective pressure on variants. Some gut metabolites vary by age, for example, the gut-microbiome-mediated bile acid profile of young children differs from adults. It could therefore be speculated that the variant specific clinical presentation of disease between children and adults may be influenced by the pediatric gut community creating a different and unstudied selective pressure based on its unique environment [91].

Moving forward, computational research is needed to model interactions between variants and important gut microorganisms and metabolites to predict variant pressures and future mutations. In addition, this will help to determine the most vulnerable populations based on their typical gut profile. 

### 2.6. COVID-19 Comorbidities Associated with Dysbiosis Imbalances in the Microbiome

The risk of severe COVID-19 illness has been associated with multiple underlying medical conditions in children [92,93,94]. Some of the most common comorbidities found amongst more severely affected children are also related to imbalances in the microbiome, such as obesity [95,96].

Children with obesity can present with factors that increase their risk of severe COVID-19, such as hypertension, insulin resistance, and high levels of proinflammatory cytokines [97,98]. Additionally, obesity is a risk factor for children with asthma that are infected with SARS-CoV-2, because it is associated with the dysfunction of their small airways [99]. These factors are problematic as there are children that have adopted less healthy lifestyles due to the pandemic and accelerated their weight gain [100,101]. Furthermore, comorbidities such as obesity, type 2 diabetes, and high blood pressure have been associated with imbalances in the gut microbiota that can result in inflammatory dysfunctions that worsen COVID-19 symptoms [102]. Animal models suggest that changes in the gut microbiota’s composition and metabolic output correlated with COVID-19-like severity in obese hamsters [103]. Moreover, alterations to specific taxa in obese hamsters were associated with pro-inflammatory parameters for the lungs and liver. However, the mechanism by which the gut microbiome in children with obesity and related comorbidities may modulate COVID-19 severity is not well understood.

In addition to obesity, there are other diseases that have been associated with pediatric gut dysbiosis that are also linked to the severity of COVID-19. For example, additional pulmonary complications that result in pneumonia in children infected with SARS-CoV-2 may also be associated with acute appendicitis, a condition associated with gut microbiome dysbiosis [104,105,106,107]. In acute myeloid leukemia (AML), a study demonstrated that the intensity of chemotherapy played a role in the immune response to COVID-19 and the severity of disease [108]. This is an intriguing connection given that AML and its treatment are associated with perturbations in the host microbiome and an overall decrease in the bacterial diversity of pediatric patients [109,110].

These multiple comorbidities related to the underlying gut microbiome provide sufficient reasoning to conduct further research into the role of the existing dysbiosis of the gut microbiome in COVID-19. Improving dysbiosis in the pediatric microbiome may decrease the risk factors for contracting severe COVID-19 infection.

## 3. Mechanistic Insights into the Role of the of Gut Microbiome in Children with COVID-19

**Figure 1:**
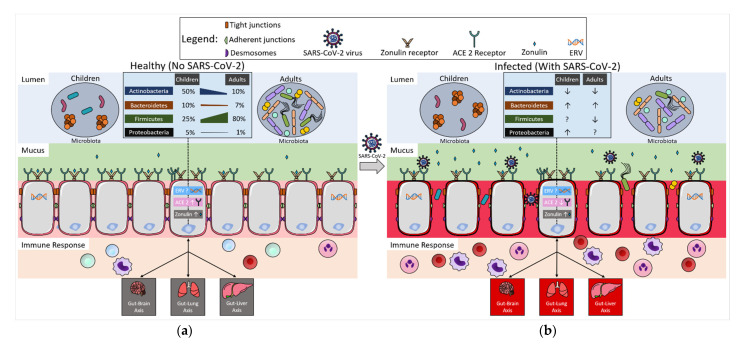
Comparison of mechanistic factors associated with the microbiome and SARS-CoV-2 response for children and adults. (**a**) Conditions associated with the gut microbiome and SARS-CoV-2 response are presented in the context of healthy children and adults. Maturation changes related to microbiome composition and bacterial diversity are demonstrated in the lumen portion. The epithelial cell barrier includes mechanistic differences in expression of angiotensin-converting enzyme II (ACE2) receptors and zonulin that can result in alterations to the immune response. In healthy conditions, children have more ACE2 receptors than healthy adults. Similarly, zonulin expression in healthy children is higher than in adults, but this is mostly reflected during the first two years of life. The impact of endogenous retrovirus (ERV) on the modulation of the gut microbiome and the immune response has not been tested yet, but it may be relevant in future studies. (**b**) Connections between the gut microbiome and SARS-CoV-2 response are shown for infected children and adults on the right side. Effects of the microbiome dysbiosis after infection with SARS-CoV-2 are highlighted, including the disruption of the epithelial cell barrier and bacterial translocation. These factors are correlated to the increases in zonulin and the decrease in ACE2 receptors observed during infection. The combination of the presented conditions and the potential role of ERV after infection could ultimately produce a greater inflammatory response. Overall, these important host-microbial events have implications for the health of other organ systems mediated through gut-axes.

Although specific results between studies varied, overall, the gut microbiome has been associated with COVID-19 in both children and adults. The developing gut microbiome of children is known to differ from the adult microbiome and educates immune health and inflammatory pathways. It is important to elucidate the mechanisms behind the bidirectional interaction between the gut microbiota and SARS-CoV-2, and how they might shape the intensity of infection and different clinical outcomes in children. Possible mechanisms of the gut microbiota that contribute to the different clinical outcomes in children and adults, including the difference in microbiota composition/metabolites, gut barrier integrity, ACE2 receptor expression, and the immune system, will be reviewed (Figure 1).

### 3.1. The Developing Microbiota in Children vs. the Mature Microbiota in Adults

It has been well documented that the gut microbiota undergoes significant changes in composition and function throughout the lifespan, with the most substantial changes in early childhood shaping the immune and metabolic systems [13]. There is rapid, dynamic development of the microbiome over the first few years of life, with increasing α-diversity [11]. A cross-sectional study performed on Japanese subjects showed that the variations between gut microbiota profiles were derived from the relative abundances of major phyla from newborns to adults: Actinobacteria (from ~50% to ~10%), Bacteroidetes (from ~15% to ~10%), Firmicutes (from ~25% to ~75%), and Proteobacteria (from ~6% to ~2%) [111]. With the maturation of the microbiota, the relative abundance of Firmicutes, the predominant phyla in adults, increased, while the others decreased. Previously, it was thought that the developing microbiota gains an adult-like configuration by three years of age, but recent studies have suggested that complete microbiota maturation may take longer [111,112,113,114,115]. Taxa from Bacteroidetes and Actinobacteria (*Bifidobacterium*) were found to be enriched in the gut microbiota of children [110,112,113,116,117], including in adolescence [118]. The numbers of taxa and functional genes are similar between healthy children and adults. However, the composition and functional potential are significantly varied [114,119]. The difference that separates older children and adult microbiota is the relative abundance of the genera, not the presence or absence of a specific genus (Figure 1a) [112]. 

Numerous studies have shown that gut microbiota composition correlates with disease severity and levels of cytokines and inflammatory markers in adult patients with COVID-19 [8,119,120]. Nevertheless, it is unclear if the variation in the microbiome at baseline prior to COVID-19 infection influenced the severity of the COVID-19 infection and the resultant microbiome. Lymberopoulos et al. examined the association between the gut microbiota and COVID-19 severity by applying a population-based association approach to 3055 healthy gut microbiome samples from adults and children over the age of two in 12 countries [121]. The 12 countries were grouped into a high-severity group and a low-severity group, according to COVID-19 infection data. At the phyla level, Actinobacteria and Firmicutes were enriched in the low-severity group, while Bacteroidetes and Proteobacteria were higher in the high-severity group (Figure 1b). *Eubacterium rectale* and *Bifidobacterium* were identified as the most beneficial taxa, which were reported to be decreased in COVID-19 patients. Moreover, *Prevotella copri*, a member of the Bacteroidetes phylum that has been associated with the development of rheumatoid arthritis, was shown to be increased in the high-severity group [121,122]. These results suggest the potential preventative resilience of the baseline microbiome of the population, and possibly a role for future preventative microbiome treatments for various disease states. Thus, it can be hypothesized that the microbiota in children may be of high preventative resilience due to features such as the relatively high abundance of Actinobacteria (*Bifidobacterium*). High preventative resilience could be achieved through inter-bacterial communication, various metabolites/products (including anti-microbial peptides and short-chain fatty acids (SCFAs)) and communication with various distal organs through gut–lung, gut–brain, gut–liver, and other axes. Further research with larger cohorts of healthy children and adults, as well as COVID-19-infected cohorts, is needed to assess this.

### 3.2. Gut Microbiota, Intestinal Inflammation, and Gut Barrier Integrity

In addition to gut microbiota alteration, intestinal inflammation, microbial translocation, and gut barrier dysfunction have also been implicated in COVID-19 patients; these may result from microbiota alterations [123]. Mucus is the first line of defense in the GI tract, physically separating environmental pathogens and antigens from the intestinal epithelium (Figure 1a). Additionally, the mucus layer provides attachment sites and nutrients to support the symbiosis of the gut microbiota [124]. Impaired mucus allows the invading pathogens to reach the intestinal epithelium, potentially contributing to inflammatory diseases [125,126]. The gut microbiota has been implicated in the formation of a proper mucus layer, evidenced by the increased penetrability of the thin mucus in germ-free mice due to the lack of gut microbiota stimulation [127]. In addition, gut microbiota composition has been shown to shape mucus properties, such as thickness and penetrability [128]. Furthermore, the bacterial products lipopolysaccharide (LPS) and peptidoglycan (PGN) may stimulate mucus secretion and restore the mucus layer of germ-free mice to the level of control mice [129]. Collectively, these bidirectional interactions between the gut microbiota and mucus layer help maintain mucosal homeostasis [130,131,132,133,134,135]. 

Underneath the mucus layer, epithelial cells form a single-layer physical barrier via tight junctions, which set limits for paracellular permeability. Zonulin is the only known regulator of intercellular tight junctions between epithelial cells. High levels of zonulin lead to increased gut permeability [136]. The secretion of zonulin by intestinal epithelial cells may be triggered by direct adherence to the apical surface of intestinal epithelial cells by bacteria, gliadin fragments, and bacteria products, such as LPS and endotoxins (Figure 1a) [137]. In children, Yonker et al. demonstrated that zonulin-dependent gut barrier dysfunction is involved in MIS-C [72]. The persistent presence of SARS-CoV-2 in the GI tract resulted in the release of zonulin, which downregulated the tight junction gene expression and led to increased intestinal permeability. The damaged barrier allowed the trafficking of SARS-CoV-2 antigens into the bloodstream, leading to hyperinflammation (Figure 1b). Treatment with larazotide, a zonulin antagonist, decreased inflammatory markers and achieved similar clinical results to current standard MIS-C treatments [72].

Increased levels of gut permeability can also lead to microbial translocation in the gut, which is the movement of bacteria and other microorganisms through the intestinal epithelial barrier into mucosal tissue and beyond. A study of COVID-19 patients (*n* = 66) demonstrated that non-survivors experienced a greater increase in LPS levels in the blood during hospitalization compared to survivors [138]. LPS is a component of the bacterial outer membrane and is indicative of the presence of bacteria through translocation. Giron et al. (*n* = 60 COVID-19, 20 healthy controls) supported this by associating high levels of zonulin in the gut, as well as LPS binding protein (LBP) and β-glucan in the blood, with severe COVID-19 and mortality, indicating that gut permeability and microbial translocation may play a role in pathogenesis [133]. Furthermore, Xu et al. clustered pediatric nasopharyngeal and stool microbiota samples into patterns to track shifts in the severity of dysbiosis during COVID-19 infection (*n* = 9) (Table 1) [138]. On post-diagnosis day 27, one stool sample resembled the pattern of dysbiosis observed in the nasopharyngeal samples, suggesting a potential translocation event. Bernard-Raichon et al. found that bloodstream infections (BSI) (*n* = 25 COVID-19) were negatively related to *Faecalibacterium*, a genus known to generate SCFAs and support gut barrier integrity [139]. They also demonstrated that BSI-causing microbes may be identified in analogous stool samples, indicating bacterial translocation in COVID-19 due to increased gut permeability during infection (Figure 1b). In addition, gut dysbiosis and microbial translocation have been postulated to play a role in long COVID through persistent inflammation [16,140,141]. 

Fecal calprotectin, a marker of intestinal inflammation, has been shown to be increased in COVID-19 infections [129]. Increased fecal calprotectin is associated with higher levels of microbiome dysbiosis, indicating a role of gut-microbiome-associated intestinal inflammation in COVID-19 [130]. Interestingly, however, increased calprotectin levels in COVID-19 did not correlate with GI symptoms [129]. Increased levels of intestinal inflammation can also lead to microbial translocation in the gut, which is the movement of bacteria and other microorganisms through the intestinal epithelial barrier into mucosal tissue and beyond. The presence of bacteria and non-indigenous microorganisms in this sterile environment triggers an immune response, and continued exposure can result in prolonged damaging inflammation [131]. The gut microbiota and immune response is heavily implicated with gut barrier integrity. While the bidirectional relationship is not fully characterized, there is concern that COVID-19 infection can further gut permeability, putting patients at higher risk for microbial translocation. This, in turn, can increase COVID-19 severity, feeding into a cycle of worsening dysbiosis, translocation, and COVID-19 pathogenesis [132,133,134].

### 3.3. Interaction between ACE2 and Gut Microbiota in COVID-19 Infection

Angiotensin-converting enzyme 2 (ACE2), the receptor for SARS-CoV-2 to enter the host cells, is highly expressed in various human organs, including the lung and gut. The expression level of ACE2 is known to be higher in the GI tract than in the lungs [142,143]. Moreover, ACE2 expression in the small intestine was 2.5-fold higher in children compared to adults (Figure 1) [143]. This may explain previous findings that GI symptoms manifest in children with COVID-19 more than they do in adult patients [144]. Additionally, ACE2 functions as a chaperone protein of B^0^AT1 and regulates the uptake of dietary amino acids, such as tryptophan [145,146]. Tryptophan preserves the symbiotic gut microbiota composition via improving gut barrier function, reducing proinflammatory cytokines, and inducing the release of antimicrobial peptides [147,148]. ACE2-deficient mice showed microbiota alterations, low plasma levels of tryptophan, and increased susceptibility to chemical-induced colitis [146]. *Bacteroides* spp. (*B. dorei*, *B. thetaiotaomicron*, *B. massiliensis*, and *B. ovatus*) were shown to downregulate ACE2 expression in the mouse intestine [149]. Interestingly, these Bacteroidetes members were reported to be inversely correlated with SARS-CoV-2 viral load in patient fecal samples [150]. ACE2 has been shown to be downregulated in COVID-19 infections [151]. Moreover, ACE2 expression in Caco-2 cells was downregulated by *A. muciniphila*, *F. prausnitzii*, *B. thetaiotaomicron*, and *B. fragilis*, and their postbiotics [152]. 

As an angiotensin-converting enzyme, ACE2 can convert Ang-I and Ang-II to angiotensin-(1–7). Angiotensin-(1–7) binds to the MAS receptor to counteract the pathophysiological effects mediated by Ang-I and Ang-II (renin-angiotensin system), including vasoconstriction, fibrosis, and inflammatory responses [153]. In addition to the direct viral effects and immune responses, the imbalance between the Ang-I/Ang-II signaling pathway and angiotensin-(1–7)/MAS axis induced by the SARS-CoV-2-mediated downregulation of ACE2 may also lead to the multiple organ injuries seen in COVID-19 [151,154]. In the gut, more Ang-II due to decreased ACE2 resulted in enhanced permeability, leading to “leaky gut syndrome” [155].

Taken together, the differences between pediatric and adult populations in the expression of ACE2 and the interaction of ACE2 with the gut microbiota may contribute to the varied clinical outcomes. A better understanding of the interaction between ACE2 and different gut microbiota communities is crucial for developing age-appropriate therapeutic strategies and preventive measures against COVID-19.

### 3.4. Gut Microbiota and the Immune Response through Endogenous Retroviruses (ERVs)

The differences in the immune responses of children and adults have been well reviewed elsewhere [73,156]. In addition to the known data, we hypothesize a possible role of endogenous retroviruses (ERVs) in mediating the communication between the gut microbiota and the immune system during COVID-19 infection (Figure 1b). Lima-Junior et al. reported that the skin microbiota may induce the expression of ERV in keratinocytes, which triggers an antiviral immune response [157]. This response supports tissue homeostasis by favoring the generation of beneficial commensal-specific T-cell subsets under healthy conditions [157,158]. Furthermore, with systemic inflammation, the enhanced ERV expression led to a dysregulated immune response and ultimately resulted in tissue pathology [157,158]. This groundbreaking research sheds light on the field of host–microbiome interactions. Given the critical role of the gut microbiota in the immune system, it can be hypothesized that the developing microbiota in children and the mature microbiota in adults may activate distinct ERV expression patterns in gut epithelial cells with different expression patterns favoring different immune responses and clinical outcomes in COVID-19 infection. This is an unstudied area that warrants further investigation. 

## 4. Vaccines and the Microbiome

Vaccines are an important mechanism of immune protection against diseases, and in recent years, researchers have started to study their connection to the microbiome. Studies have shown that the gut microbiome plays a critical role in the establishment of vaccine immune response and efficacy [159]. Tests on murine models showed that TLR5-mediated sensing of the microbiota was relevant to the development of immune responses for the polio vaccine [160]. Furthermore, correlations have been identified between the gut microbiome and vaccine responses to different viruses, such as rotavirus [161,162,163]. In studies with rotavirus, the microbiome was significantly different between the group that effectively responded to the vaccine and the group that did not. However, the fact that rotavirus affects the GI tract, and that the vaccine is given orally, must be taken into consideration as the gut microbiome may have more of an impact in this instance. 

Specific phyla of bacteria have also been associated with improvements in vaccine responses [164]. For both oral and parenteral vaccines, a greater relative abundance of the phylum Actinobacteria was associated with both higher humoral and higher cellular vaccine responses, while a greater relative abundance of the phylum Proteobacteria was associated with lower responses [165]. Bifidobacterium predominance may enhance thymic development and responses to vaccines early in infancy [166,167]. Studies to determine the effects of microbiome composition on the COVID-19 vaccine have just started in adults [168]. Specific but different gut bacterial taxa were associated with a higher immune response in those receiving an inactivated vaccine versus an mRNA vaccine. Higher levels of *Bifidobacterium adolescentis* were associated with higher neutralizing antibodies for the inactivated vaccine, whereas *Roseburia faecis* was enriched in those with higher neutralizing antibodies for the mRNA vaccine.

Certain components of the healthy microbiota are important for the development of vaccine responses against respiratory viruses such as SARS-CoV-2, with gut microbiome dysbiosis potentially resulting in suboptimal responses [169,170]. This has been demonstrated in mice, where early life antibiotic-induced dysbiosis of the gut resulted in impaired antibody responses across different vaccines; this was not seen in adult mice treated with antibiotics [171]. Restoration of the commensal microbiota after the antibiotic exposure allowed for normal vaccine responses, giving hope for microbial therapies to improve vaccine responses. Although there are no existing studies to validate the effects of antibiotics on the vaccine response of infants, adults have demonstrated that perturbations in the microbiome by antibiotics led to alterations in vaccine immune responses [172]. 

The important role of the microbiome has led experts to question whether we should consider the status of the host’s microbiome before attempting to develop vaccines [173]. Development of oral vaccines and maintenance of the microbiota may help in the early control of COVID-19 or other outbreaks.

Along this line, researchers have started to give more consideration to possibly using the microbiome as adjuvants for vaccines [174]. Tests for the effectiveness of combining the microbiome and vaccines can be traced back to 1995, when Isolauri combined a *Lactobacillus casei* strain GG with a rotavirus vaccine [175]. This combination resulted in an immunostimulating effect in rotavirus vaccination. However, even after years of studies, there is still a large gap in knowledge regarding the effects that the microbiota may have in vaccination when applied as an adjuvant [176,177].

Similarly, researchers have tried to use probiotics in conjunction with vaccines to boost the process of immunization [178]. Early introduction of probiotics may provide significant beneficial immune outcomes in neonates prior to commencing a vaccination schedule [179]. One study showed that using *Lactobacillus* as a probiotic with vaccines improved the capacity of infants to mount immune responses against protein agents [180]. Probiotics may also have immunomodulatory effects on vaccine responses for children whose mothers had a history of allergic disease [181]. Recently, a murine study indicated that a probiotic strain of *Lactobacillus plantarum* improved immune response to SARS-CoV-2 vaccination [182].

Further understanding of how the gut microbiome modulates the SARS-CoV-2 vaccine response in humans, using novel multi-omic techniques, may lead to increased responses using adjuvant microbial therapies [183,184]. It is also essential to study pediatric cohorts as the gut microbiome of a young child differs significantly from the adult microbiome, and results from adult studies may not be directly applicable. Moreover, early life microbiome development educates the immune system, so the impact of the baseline microbiome and microbial adjuvants in vaccines may be more pronounced in children. However, there is concern regarding the alteration of the microbiome during this critical period of development.

## 5. Probiotics and Other Microbial Therapeutics in COVID-19

There are many microbial therapeutics with the potential to support development of a healthy gut community, including probiotics, prebiotics, synbiotics, specific dietary interventions, and fecal microbiota transplantation (FMT). Probiotics are generally defined as live microorganisms used to promote human microbial health. While their role in different areas of medicine is still being determined, it is known that they may promote a healthier microbial space through competition and other roles [185]. Prebiotics work similarly to probiotics but are composed of oligosaccharides that encourage growth of beneficial bacteria in the host [186]. Synbiotics have live microorganisms and microbe-supporting compounds, making them a synergetic combination of both [187].

Oral microbial therapeutics have been used in pediatric and adult populations to reduce the occurrence and severity of respiratory infections. They can exhibit antiviral effects by strengthening the gut–lung axis and regulating the host inflammatory response [188].

In a pediatric trial (*n* = 31 prebiotic, 31 probiotic, 32 placebo), a prebiotic blend of galactooliggsaccharide and polydextrose or the probiotic *Lactobacillus rhamnosus* GG was administered with breast milk during the first 60 days of life in preterm infants [189]. The prebiotic and probiotic cohorts saw reduced instances of virus-associated respiratory tract infections (*p* < 0.001 and *p* = 0.022, respectively). A study administering Lab4P probiotic to overweight and obese adults (*n* = 220) daily suggested that probiotics may reduce upper respiratory tract infection symptoms and stabilize gut microbiota diversity, which may be particularly relevant in COVID-19 infections, in which obesity is associated with worse outcomes [101,190]. 

Despite the potential, there is limited research using oral microbial therapeutics to treat COVID-19 in pediatric populations. Although there have only been a few trials of probiotics in adults with COVID-19 infection, there have been promising results, demonstrating changes in mortality, occurrence of diarrhea, hospitalization time, antibody formation, and viral load (Table 2) [191,192,193,194,195,196]. A booster oral dose of the *Bifidobacterium animalis* sp. *Lactis* strain was administered to adults with moderate/severe COVID-19 (*n* = 20 probiotic, 24 non-probiotic) (Table 2) [193]. The probiotic group had five days reduced hospitalization time (*p* < 0.001) and decreased IL-6 levels (*p* < 0.001). A larger prospective, randomized-controlled trial of a multi-strain probiotic in adults with COVID-19 (*n* = 99 probiotics, 101 controls) showed a decreased frequency in developing diarrhea with probiotic treatment compared to those receiving a single dose of antibiotics, but no difference in mortality rates or most biomarkers (Table 2) [192]. The probiotic *L. fermentum* 90 TC 4 demonstrated antiviral properties against SARS-CoV-2 (*p* < 0.005) when cultured with VERO E6 cells, indicating the potential to advance to animal models and eventually clinical trials [197]. These studies support the use of oral therapeutics to reduce frequency, duration, and risk of viral respiratory infections. However, oral therapeutics have yet to become a standard part of prevention and treatment due to other conflicting studies finding that changes in mortality, biomarkers, microbiota composition, and oxygen requirement are not significant in oral microbial-therapeutic-treated groups (Table 2) [192,194,196]. Additionally, a meta-analysis of 23 trials including children and adults (*n* = 6950) found that there is low to very low certainty that probiotics may reduce the number of people affected, the frequency of upper respiratory tract infections, and their duration, requiring more conclusive research in the future [198]. They did report that the adverse events for probiotic use were minor and GI related, and that there were no statistically significant differences in the occurrence between probiotic and placebo groups, suggesting that even if there is questionable benefit, probiotics are safe to use. 

Furthermore, oral microbial therapeutics have been used to prevent and treat sepsis, an extreme and fatal immune response to infection. In a clinical trial (*n* = 2278 synbiotic, 2278 control), a synbiotic blend of *Lactobacillus plantarum* and fructooligosaccharide was administered to newborns daily for a week in rural India [199]. After 60 days of observation, the synbiotic cohort saw a significant reduction in death and sepsis compared to the control group. A clinical trial (*n* = 50 probiotic, 50 control) with children of ages ranging from 3 months to 12 years with severe sepsis treated patients daily for a week with the probiotic VSL#3 [200]. This eight-strain blend included *Bifidobacterium longum, B. infantis, B. breve,* and *Streptococcus salivarius*. The groups had similar cytokine levels before treatment. After treatment, the probiotic group had lower levels of proinflammatory cytokines (IL-6, IL-12p70, IL-17, TNF-α) and higher levels of anti-inflammatory cytokines (IL-10, TGF-β1) compared to the placebo group, but with minimal effects on clinical outcomes. Interestingly, the probiotic used in that study included *S. salivarius,* which is hypothesized to fortify the oral and lung microbiota, protecting from severe COVID-19 infection or co-infection from dysbiosis [201]. In a clinical trial, patients with sepsis ventilated in the intensive care unit (*n* = 35) were administered a synbiotic of *Bifidobacterium breve* strain Yakult, *Lactobacillus casei* strain Shirota, and galactooligosaccharides [202]. The rate of infectious complications was less than half in the synbiotic group (*p* < 0.05). The rate of enteritis and ventilator-associated pneumonia (VAP) was significantly lower in the synbiotic group (*p* < 0.05). Moreover, a computational analysis of bacteriocin products of the common probiotic *Lactobacillus plantarum* found that some of the plantiricin compounds may competitively inhibit binding with RdRp, RBD, and ACE2 proteins of SARS-CoV-2, indicating potential for a *L. plantarum* probiotic treatment for COVID-19 [203]. These previous studies suggest a possible role for oral therapeutics in reducing the severity of pediatric COVID-19 infection and increasing healthy immune responses.

Diet is another known factor that shapes the gut environment as it supplies nutrients, as well as natural prebiotics and probiotics [204]. The western diet, known for its high intake of refined carbohydrates and saturated fats, has been correlated with heighted immune activation and inflammation, which may contribute to the severity of COVID-19 [205]. The NurtiNet-Santé cohort (*n* = 7766) revealed that those with higher dietary intake of certain vitamins and fiber via fruits and vegetables were less susceptible to COVID-19 infection [206]. Additionally, a clinical study of antibiotic-naïve patients (*n* = 66) showed that the microbial pathways were altered during COVID-19 infection, resulting in depleted carbohydrate degradation capacity and increased urea cycle activity [207]. Furthermore, greater severity of disease correlated with reduced sugar derivation and increased carbohydrate biosynthesis, suggesting that there are significant changes in the microbial environment and pathways due to COVID-19 infection that may influence host immune response and metabolism. This increases the need for research regarding the ideal nutrition during COVID-19 infection, as well as post-recovery treatments to recoup the function of these important microbial pathways. Indeed, “immunonutrition” has been suggested as a strategy in children with obesity to reduce the severity of SARS-CoV-2 infection, but no clinical trials have addressed this yet [108]. Specifically for infants, breast milk, which in addition to being the optimal source of nutrition in early life is a known prebiotic and probiotic, conveys protection against respiratory infections in the first year of life. In a population-based prospective study of infants that were never breastfed (*n* = 519), breastfed for <4 months (*n* = 1203), for 4–6 months (*n* = 1012), and ≥6 months (*n* = 1404), there was a significant decrease during the first 6 months of life in upper and lower respiratory tract infections (*p* < 0.01 and *p* < 0.05, respectively) in infants breastfed for at least 6 months [208]. Further, there was a significant decrease during 7–12 months of age in lower respiratory tract infections for those breastfed for at least four months (*p* < 0.1). There is evidence that breastfeeding also protects against COVID-19 in infants, although it is difficult to know what component of breast milk provides protection as antibodies to SARS-CoV-2 from maternal infection or vaccination can also be detected in breast milk [209,210,211].

Additionally, SCFAs formed by fermentation of fiber and complex carbohydrates are considered indicative of good gut health. Research has shown that higher levels of SCFAs in stool at one year old was associated with lower occurrences of atopy, asthma, allergic rhinitis, and food allergies [17]. Furthermore, reduced levels of SCFA-producing bacteria were associated with multiple sclerosis, and alterations in SCFA levels were associated with type 1 diabetes. It has been hypothesized that SCFAs, specifically butyrate, can be used to modulate immune response and prevent cytokine storms in severe instances of COVID-19 [212]. Using a SARS-CoV-2 pseudovirus in a murine model, SCFAs were demonstrated to reduce viral entry and promote antiviral immunity [213]. Both adult and pediatric COVID-19 studies have demonstrated reduced levels of *F. prausnitzii*, a key synthesizer of SCFAs, in the gut [16,150,207,214]. However, there were no significant differences in the SCFA levels between probiotic and control groups in a study mentioned above [202], suggesting that diet-based changes may be the best mechanism to support the gut and generation of SCFAs.

Finally, fecal microbiota transplant (FMT) is the transfer of a healthy microbial community from a donor to a recipient with an altered gut microbiome. It is an effective treatment for *Clostridioides difficile* infection (CDI) in both adults and children [215,216] with the potential to treat a variety of illnesses with underlying gut microbiome dysbiosis, including COVID-19. Two cases have been described with COVID-19 occurring shortly after patients were treated with FMT for CDI [217]. The first patient was an 80-year-old with comorbidities, and the second patient was a 19-year-old on immunosuppression medicines. Both patients and their fecal transplant matter tested negative for SARS-CoV-2 prior to FMT procedure. However, the CDI patients tested positive for COVID-19 post-procedure. The 80-year-old patient received remdesivir and convalescent plasma (CP). Both patients were at high risk for severe COVID-19 progression, but only experienced mild progression. The first patient saw clinical improvements two days after the FMT and COVID-19 treatment began, although it typically takes ten days for clinical benefits to begin. These findings suggest that the FMT may have helped mediate the immune response during COVID-19 infection before the standard care treatments could take effect. Some suggest using convalescent fecal microbiota for FMT as opposed to healthy donors, but this raises concerns for the transfer of fecal matter with persistent dysbiosis [218]. Overall, more research is required to fully examine the role of FMT in COVID-19 treatment. An active clinical trial (Identifier No. NCT04824222) aims to understand the potential of FMT to reduce the risk of COVID-19 progression and complications [219]. Others have published a protocol to test the efficacy of washed microbiota transplantation (WMT), a more processed version of FMT, for COVID-19 patients [220]. Nevertheless, there is concern about the potential transmission of SARS-CoV-2 with FMT given the virus can be detected in the stool; therefore, all donors and FMT material should be screened for SARS-CoV-2 [221,222]. Due to this concern and other factors, including lack of access to screened FMT material, FMT rates for CDI in children decreased significantly during the first year of the pandemic, so there is currently no data available on the effects of FMT in children with co-existing CDI and COVID-19 [223,224].

## 6. Effect of Pandemic as a Whole on the Gut Microbiome (i.e., the Hygiene Hypothesis)

The COVID-19 pandemic may have implications on the human microbiome of individuals, even if they have not been infected. The effects of the pandemic on the human microbiome have been referred to in other sources as part of the “hygiene hypothesis”. Finlay et al. explained that measures to avoid viral infection have led people to have less interactions with each other, and that, in conjunction with increases in their hygiene methods, can potentially impact the microbiome [225]. Human interactions are essential to increase and sustain the diversity of our gut microbiota. For example, studies have found that close relationships between individuals led to microbial communities with greater diversity and richness relative to those living alone. Furthermore, a study demonstrated that children living on farms with animals had a wider range of microbes and fewer risks of developing diseases like asthma [226]. This means that not only interactions with humans, but interactions with animals and our environment in general are important to mature our microbiome. Eventually, these long-term consequences in the human microbiome as a result of the COVID-19 pandemic may not only affect susceptibility to SARS-CoV-2 but also lead to diseases associated with microbiome abnormalities [227]. This may be especially important for young children during their critical window of microbiome development early in life, which is crucial for immune education. Conversely, antibiotic use, which can have a profound negative impact on the developing microbiome and is associated with obesity and other inflammatory diseases later in life, was found to be reduced in children, especially during the start of the pandemic, which may possibly counteract some of these effects [228]. 

It is unclear at this point what impact the lack of usual childhood exposures during the COVID-19 pandemic and potential changes in the microbiome will have on future health. There is concern that an increase in atopic and autoimmune diseases may be seen later in life for those who were infants and young children at the start of the pandemic, who may have altered early life microbiome development [17]. Cohorts of children born during the pandemic should be closely followed to assess these potential adverse health outcomes.

Now that vaccines are available for most people, some may argue that the extreme hygiene cycle should be over. As the pandemic is still ongoing, it is important to take protective measurements against COVID-19. With this purpose, researchers developed probiotic-based sanitation that may stably decrease pathogens on surfaces while avoiding the promotion of antimicrobial resistance for use in a children’s hospital [229]. In this way, the probiotic cleaner better conserves beneficial bacteria and stops pathogenic bacteria from acquiring mutations that improve their pathogenicity [228,229,230]. The development of alternative methods to counter the effects of the pandemic should be promoted to avoid potential changes in the microbiome that may result in the worsening of health outcomes.

## 7. Conclusions

The gut microbiome is an essential component in regulating the immune response against the SARS-CoV-2 virus. Although most children show mild symptoms or asymptomatic infection, gut microbiome dysbiosis can occur during infection, with potential long-term consequences. The mechanism for the role of the gut microbiome in COVID-19-infected children is still not well understood, but it is suggested that there is important microbial mediation of ACE2 interactions and gut barrier integrity. Further, the gut microbiota may play a key role in vaccine efficacy, as well as variant evolution. For this reason, there is a need to perform additional clinical and mechanistic studies to understand the role of the pediatric microbiome in response to SARS-CoV-2, which may differ from adults given differences in the microbiome and immune system between children and adults. This knowledge may lead to increased treatments available for children, such as microbial therapeutics, and improved health outcomes.

## Figures and Tables

**Table 2:** Current research on oral microbial therapeutics in relation to COVID-19 infection.

Title	Author, Location, Date Published	Patients	Ages	Oral Therapeutic	Duration	Outcome
Oral Bacteriotherapy in Patients with COVID-19: A Retrospective Cohort Study [191]	Ceccarelli G et al. Rome, Italy January 2021	200 hospitalized COVID-19 patients (*n* = 88 bacteriotherapy, 112 untreated)	≥18 years	Bacteriotherapy: *Streptococcus thermophilus* DSM 32245, *Bifidobacterium lactis* DSM 32246, *Bifidobacterium lactis* DSM 32247, *Lactobacillus acidophilus* DSM 32241, *Lactobacillus helveticus* DSM 32242, *Lactobacillus paracasei* DSM 32243, *Lactobacillus plantarum* DSM 32244, *Lactobacillus brevis* DSM 27961	Three times daily Duration not specified	Decreased mortality in bacteriotherapy-treated group (*p* < 0.001) Treated group had higher CRP concentrations and lower LDH, untreated group had lower albumin initially (*p* < 0.025)
Efficacy of a Probiotic Consisting of *Lacticaseibacillus rhamnosus* PDV 1705, *Bifidobacterium bifidum* PDV 0903, *Bifidobacterium longum* subsp. *infantis* PDV 1911, and *Bifidobacterium longum* subsp. *longum* PDV 2301 in the Treatment of Hospitalized Patients with COVID-19: a Randomized Controlled Trial [192]	Ivashkin V et al. Moscow, Russia October 2021	200 hospitalized COVID-19 patients (*n* = 99 probiotic, 101 nonprobiotic)	18–75 years	Probiotic: *Lacticaseibacillus rhamnosus* PDV 1705, *Bifidobacterium bifidum* PDV 0903, *Bifidobacterium longum* subsp. *infantis* PDV 1911, *Bifidobacterium longum* subsp. *longum* PDV 2301	Three times daily For up to 14 days ending sooner if discharged or dead	Decreased length of COVID-19-associated diarrhea (*p* = 0.049) Decreased occurrence of hospital-acquired diarrhea in probiotic patients treated with single antibiotic (*p* = 0.023) Changes in mortality and biomarkers were not significant
Oral booster probiotic bifidobacteria in SARS-CoV-2 patients [193]	Bozkurt H et al. Istanbul, Turkey November 2021	44 hospitalized COVID-19 patients (*n* = 20 probiotic, 24 nonprobiotic)	≥18 years	Probiotic: *Bifidobacterium animalis* subsp. *Lactis* BB-12	Daily For 3 days	Hospitalization time in probiotic group reduced by 5 days (*p* < 0.001) Decreased IL-6 levels in probiotic group (*p* < 0.005) Statistical analysis limited by small cohort sizes No demographic or baseline characteristics compared between groups
Probiotic improves symptomatic and viral clearance in COVID-19 outpatients: a randomized, quadruple-blinded, placebo-controlled trial [194]	Gutiérrez-Castrellón et al. Mexico City, Mexico January 2022	293 symptomatic COVID-19 patients (*n* = 147 probiotic, 146 placebo)	18–60 years	Probiotic: *Lactiplantibacillus plantarum* stains KABP022, KABP023 and KABP033, *Pediococcus acidilactici* strain KABP021	Daily For 30 days	Increased occurrence of complete viral and symptom remission in probiotic group (*p* < 0.001) Reduced nasopharyngeal viral load on days 15 and 30 in probiotic group (*p* < 0.001) Increased SARS-CoV-2-binding IgG and IgM in serum (*p* < 0.001) Changes in microbiota compositions were not significant
Gut microbiota-derived synbiotic formula (SIM01) as a novel adjuvant therapy for COVID-19: An open-label pilot study [195]	Zhang L et al. Hong Kong, China March 2022	55 COVID-19 hospitalized patients (*n* = 25 probiotic, 30 nonprobiotic)	≥18 years	Synbiotic: *Bifidobacterium adolescentis*, *Bifidobacterium bifidum*, *Bifidobacterium longum*, galactooligosaccharides, xylooligosaccharide, resistant dextrin	Twice daily For 28 days	Increased occurrence of IgG antibody formation by day 16 in probiotic group (*p* = 0.037) Reduced levels of IL-6, M-CSF, TNF-α, and IL-1RA at week 5 in probiotic group (*p* < 0.01) Increased abundance of Actinobacteria and Firmicutes, decreased abundance of *E. coli* and *Bacteroides* spp. at week 5 in probiotic group (*p* < 0.05) No significant abundance increases for probiotic strains in treated group
COVID-19 Pneumonia and Gut Inflammation: The Role of a Mix of Three Probiotic Strains in Reducing Inflammatory Markers and Need for Oxygen Support [196]	Saviano A et al. Rome, Italy July 2022	80 hospitalized COVID-19 patients with interstitial pneumonia (*n* = 40 probiotic, 40 nonprobiotic)	≥18 years	Probiotic: *Bifidobacterium lactis LA 304*, *Lactobacillus salivarius LA 302*, *Lactobacillus acidophilus LA 201*	Twice daily For 10 days	Reduced fecal calprotectin levels in probiotic group at both time points during treatment (*p* = 0.005, *p* = 0.006) Oxygen requirement reduction is not statistically significant
